# The power of moving fast: responsible leadership, psychological empowerment and workforce agility in energy sector firms

**DOI:** 10.1016/j.heliyon.2022.e11188

**Published:** 2022-10-20

**Authors:** Szymon Cyfert, Witold Szumowski, Wojciech Dyduch, Maciej Zastempowski, Paweł Chudziński

**Affiliations:** a Institute of Management, Poznań University of Economics and Business, al. Niepodległości 10 Street, 61-875 Poznań, Poland; b Wroclaw University of Economics and Business, Faculty of Management, Komandorska 118/120, 53-345, Wroclaw, Poland; c Faculty of Economic Sciences and Management, Nicolaus Copernicus University, Gagarina 13A Street, 87-100 Toruń, Poland; d Faculty of Management, University of Economics in Katowice, ul. 1 Maja 50, 40-287 Katowice, Poland; e Poznań University of Economics and Business, al. Niepodległości 10 Street, 61-875 Poznań, Poland

**Keywords:** Responsible leadership, Organisational agility, Workforce agility, Psychological empowerment, Energy sector firms

## Abstract

The energy sector is undergoing significant transformation induced by environmental changes and increasing pressure from stakeholder groups. In order to quickly seize opportunities in the unpredictable contemporary business environment, leaders increasingly face the challenge of ensuring an appropriate level of organisational agility, achieved through workforce agility. In striving to achieve workforce agility, responsible leaders should consider the intrinsic motivation oriented towards work, how it affects a team's performance, and the level of its involvement. Based on studies that combine leadership, empowerment, and agility, we analyse whether and how responsible leadership and psychological empowerment support workforce agility in the energy sector firms. Using structural equation modelling, we analyse data gathered from a group of 187 managers and experts. The results support a hypothesised relationship between leadership focused on responsible management, psychological empowerment, and workforce agility. The survey reveals that a combination of responsible leadership and psychological empowerment affects workforce agility.

## 1 Introduction

One of the current challenges faced by companies in the energy sector is the increasing pace and complexity of technological innovation [1], the fragmentation of markets [2], increased social [3] and customer expectations [4], which translates into increasingly unpredictable and dynamic environments [5]. Arguing that the global energy sector is plunged into chaos, scholars highlight the rising energy demand, global economic crises, climate change policies, peak oil phenomena, geopolitical tensions, the collapse of nuclear energy and falling costs of renewable energy technology [6]. In such a quickly and constantly changing environment, organisations that strive to develop and increase firm performance need to ensure a high level of agility [7, 8, 9, 10]. It is posited that companies with higher levels of agility deal with rapid changes and multi-level cross-relationships more effectively, achieve greater business outcomes, and create value for their stakeholders faster [11]. There is a notion that agility can help companies achieve high customer responsiveness, as well as successfully and effectively manage market changes to reduce operational uncertainty and improve competitive advantage [12]. It should be noted that organisational agility cannot be achieved without an agile workforce, as workforce agility contributes to a high level of quality, better customer service, acceleration of the learning curve and economies of scale [13]. Al-Faouri et al. extend the discussion by suggesting that organisations cannot be efficient without workforce agility since they are employees, not machines, that anticipate change and contribute to the success of an organisation through their knowledge, ideas, judgment and cooperation [8], which supports the argument to nurture agility at the employee level [14]. Researchers also suggest that developing the workforce agility requires responsible leadership, which plays a significant role in empowering employees, creating an agile culture [11, 15] as well as stimulating collaboration and orientation on people [16]. Although leaders at various levels of organisations play a key role in shaping relationships with stakeholders [17], it should be noted that a CEO's responsibility – a key ingredient of leadership, does not boil down to giving power to employees, but concerns verifying whether employees actually feel empowered [18]. Empowerment within the work environment has two different perspectives: structural empowerment (workplace) and psychological empowerment (worker's cognitions [19]). While structural empowerment focuses on providing access to information and resources, supporting activities and providing opportunities for learning [20], psychological empowerment refers to empowerment on an individual level [21], to the worker's beliefs regarding their competence and autonomy, and the outcomes that their work provides to the organisation [22]. Responsible leaders should empower people by assigning power and autonomy as they perform their roles in the workplace [23].

The paper is structured as follows. The conceptual framework is described in section 2, the methods are outlined in section 3, and the results are presented in section 4. Section 5 discusses the results, while conclusions are presented in section 6.

## 2 Literature review and hypotheses development

### 2.1 Responsible leadership

In defining responsible leadership, Haque et al. show that the notion of responsible leadership has emerged from studies at the intersection of ethics, leadership and corporate social responsibility [24]. Embedding the discussion on responsible leadership in the theory of stakeholders and social responsibility, Maak and Pless emphasise that responsible leadership is a socio-relational and ethical phenomenon that appears in the processes of social interactions [25].

Maak et al. indicate that while there are different understandings of responsible leadership, interactions with stakeholders are an important part of responsible leadership [26]. Also, Javed et al. show that responsible leadership considers leadership as a leader-stakeholder multivalent relationship, whereas earlier forms treat leadership as a leader-follower dyadic equation [27]. Meanwhile, Sarkar points out that responsible leadership has the essential characteristics of three leadership styles - transformational, servant and authentic [28]. Maak emphasises the importance of responsible leadership in creating social capital and sustainable business [29]. Miska et al. pay attention to the challenges, pressures and complexities of responsible leadership in finding the balance between maximising profits and living up to responsibilities [30].

Emphasizing the importance of interactions with stakeholder groups, Maak et al. assume that responsible leadership is a relational process of interaction between leaders and stakeholder groups aimed at defining responsibilities in matters related to creating organisational value [26]. Assuming that responsible leadership forces leaders to open up to a wider target stakeholder groups, Voegtlin et al. indicate that responsible leadership includes activities related to considering the consequences for all stakeholder groups, influencing by enabling engagement of stakeholder groups, and engaging in active dialogue with stakeholder groups [31].

### 2.2 Workforce agility

Storme et al. suggest that workforce agility has long been recognised as a foundation of organisational agility. Without a workforce that is both willing and able to adapt to change, any strategy for implementing new ways of working is doomed to failure [32]. Breu et al. point to the importance of an agile workforce as a key force in creating an agile organisation, which can quickly respond to changes in the turbulent environment [33]. Meanwhile, Qin and Nembhard suggest that there is a general rule agreed upon in agility literature that workforce agility is an essential aspect of the overall agility of an organisation [34]. Al-Faouri et al. note that employee flexibility is defined as the ability of employees to solve problems actively, strategically respond to uncertainty and change business needs using their diverse business and technical knowledge [8]. Sherehiy and Karwowski reject the perception of workforce agility in terms of agile personality, predisposition or attributes, and define it as observable agile performance or behaviours [13].

### 2.3 Psychological empowerment

Achieving success requires the involvement of all employees in the processes of adjusting the organisation to the changing environment. Uner and Turan suggest that the best-in-class organisations achieve this goal by empowering employees to take initiative without provoking, serving the corporate interests without micromanaging, and acting like company owners [35].

Siegall and Gardner suggest that empowered employees perceive themselves as more effective and are judged by their co-workers as more effective [36]. Similarly, Sun et al. argue that a high level of psychological empowerment is associated with the ability to cope with organisational stressors [37]. Also, Li et al. note a relationship between high levels of psychological empowerment and low stress, burnout and turnover, and high organisational commitment and job satisfaction [19].

Table 1 shows a synthesis of the approaches to define the described constructs.

**Table 1:** Synthesis of the approaches to define the described constructs.

Author	Attributes
Responsible Leadership	
Voegtlin et al. [38]	(1) Awareness of relevant stakeholder claims; (2) Awareness of consequences of decisions for the affected stakeholders; (3) Involving the affected stakeholders in the decision making process; (4) Taking into account different stakeholder claims before making a decision; (5) Achieving a consensus among the affected stakeholders.
Maak [29]	Ability involved in: (1) building, (2) cultivating and (3) sustaining trustful relationships to different stakeholders, both inside and outside the organization, and in (4) co-ordinating responsible action to achieve a meaningful, commonly shared business vision.
Doh et al. [39]	(1) Stakeholder Culture (five items; e.g., “I like to understand how things work”); (2) Human Resource Practices (five items; e.g., “Our performance appraisal programs are effectively used to retain the best talent”); (3) Managerial Support (four items; e.g., “My immediate manager leads by example”).
Workforce Agility	
Breu et al. [33]	(1) Responsiveness to changing customer needs; (2) Responsiveness to changing market conditions; (3) Speed of developing new skills and competencies; (4) Speed of acquiring the skills necessary for business process change; (5) Speed of innovating management skills; (6) Speed of acquiring new management skills; (7) Effectiveness of cooperating across functional boundaries; (8) Ease of moving between projects; (9) Employee empowerment for independent decision making; (10) Support for the introduction of new management systems and methods.
Storme et al. [32].	(1) Job-related curiosity (eight items; e.g., “I like to understand how things work”); (2) Job self-efficacy (seven items; e.g., “I complete successfully my tasks at work”); (3) Learning from past mistakes (six items; e.g., “I feel I have learned a lot from my mistakes”); (4) Anticipation and planning (six items; e.g., “Before embarking on a project, I try to consider several different action plans”); (5) Active listening (six items; e.g., “I am interested in listening to what others can teach me”); (6) Risk-taking (six items; e.g., “I am willing to invest my time in a project I believe in, even if it is risky”); (7) Trust (six items; e.g., “I do not suspect motives hidden in people with whom I work”); (8) Ambiguity tolerance (six reversed items; e.g., “I need information to be presented clearly to be comfortable”).
Faouri et al. [8].	(1) The ability to adapt quickly to unexpected changes; (2) The ability to learn to respond to new market requirements; (3) the ability to function efficiently under stressful conditions in a changing environment.
Muduli [9]	(1) Comfort in face of change, new ideas and new technologies; (2) Flexibility to quickly from task to task, job to job, and place to place; (3) Skill mapping, benchmarking for skill assessment and skills development; (4) Comfort in face cross-functional project teams, collaborative ventures with other companies, or with a virtual organization; (6) Tech-savvy and knowledge in advanced manufacturing technologies, IT skills, use of mobile technologies, etc.; (7) Developing skills quickly, adapting to new environments and collecting information; (8) Interest in collecting information about the organization and other related organizations.
Sherehiy and Karwowski [13]	(1) Proactivity; (2) Adaptivity; (3) Resilience.
Psychological empowerment	
Stewart et al. [40]	Sense of motivation in relation to the workplace environment.
Thomas and Velthouse [41]	Intrinsic task motivation reflecting (1) a sense of control in relation to one's work and (2) an active orientation to one's work role.
Spreitzer [42]	(1) Meaning, (2) Competence, (3) Self-determination; (4) Impact.

### 2.4 Responsible leadership and workforce agility

Arguing that leadership plays a central role in creating agile organisations, Joiner suggests that leaders should develop proactive practice in the contexts of leading organisational change, leading teams and holding pivotal conversations [11]. Pointing to the advisability of researching the relationships between leadership and workforce agility, Petermann and Zacher suggest that leadership plays a great role in initiating collaboration between teams and departments, and that leaders play a significant role in empowering employees and creating an agile culture [15]. A Varshney and Varshney survey shows that workforce agility in SMEs requires leadership that is collaborative, people-oriented and focused on self-organising teams with interchangeable roles and responsibilities [16].

We suggest that responsible leadership can facilitate workforce agility by emphasizing interaction with stakeholders [29], influencing flexibility and participation [28], and ensuring an exchange between leaders and stakeholders [43]. We believe that in order to build relationships with stakeholders, who sometimes have contradictory and rapidly changing goals, employees are needed who are adaptable and flexible [16], demonstrate the ability to react to a turbulent and changing environment [32, 44], can troubleshoot problems day-to-day [8], are able to learn to be responsive to new market demands, and are able to function efficiently under stress [8]. Thus, based on the above studies, we propose the following hypothesis:

Hypothesis 1 Responsible Leadership has a direct positive effect on Workforce Agility.

### 2.5 Responsible leadership and psychological empowerment

The close association between various leadership styles and psychological empowerment has been shown in numerous organisational studies [18, 45, 46, 47]. Houghton and Yoho suggest that the specific approach to leadership influences a certain combination of predicted outcomes: the level of follower involvement, dependence, creativity and psychological empowerment [48].

The number of studies showing how leadership can influence the sense of motivation related to the work environment can be taken as a measure of the importance of the problem [49, 50, 51]. It is worth noting, however, the lack of empirical evidence regarding the relationships between responsible leadership and psychological empowerment, as the above studies are limited to other types of leadership. Pless and Maak suggest that responsible leaders train and empower employees to achieve goals in an ethical, respectful, and ‘relatively intelligent’ manner by creating a system of incentives for respectful collaboration in order to support stakeholder responses [25]. Also, Haque et al. highlight the positive influence of responsible leadership on the organisational commitment of employees [24]. Antunes and Franco show that responsible leaders have a strong sense of the importance of the needs and interests of stakeholders that may be influenced by their actions and decisions, which makes managing relations with employees in organisations especially important [52].

By enhancing responsibility [53], influencing the building of social capital and sustainable business [29], ensuring learning agility [28], the balance between profit maximisation and acceptance of social or environmental responsibility [30], shaping relationships and creating leadership skills within stakeholder groups [54], responsible leaders influence employees and instil in them intrinsic task motivation and confidence in achieving targets successfully. We therefore hypothesise that:

Hypothesis 2 Responsible Leadership has a direct positive effect on Psychological Empowerment.

### 2.6 Psychological empowerment and workforce agility

Storme argues that the way to overcome resistance to change is to understand the psychological characteristics of agile workers. Knowledge of the determinants of potential agility allows this potential to be strengthened and workforce agility to be increased [32]. Pointing out that empowerment and autonomy in decision making are seen to be key in making a workforce truly agile, Muduli suggests that psychological empowerment is a determining factor in the direction and power of the influence of organisational practices on workforce performance [55]. Mudula's subsequent research also found that agility is supported by psychological empowerment, leading to self-determination, meaning, and competence [9]. Muduli and Pandya show that psychological empowerment, namely meaningfulness, competence, self-determination and impact, influence workforce agility [10]. By suggesting that agile behaviour is openly linked to intrinsic motivation and high job satisfaction, Paul et al. assume that when employees feel from within that they are making a difference to the organisation through their work, then this results in augmenting workforce agility [14]. Also, the findings of Almahamid show that workers' psychological empowerment has a positive effect on the ability to be agile [56], although the results of a study by Malik et al. suggest that agile practices related to team diversity and incremental and iterative development were not found to have a valid relationship with psychological empowerment [57].

Based on such findings, the following hypothesis can be proposed for our study context:

Hypothesis 3 Psychological Empowerment has a direct positive effect on Workforce Agility.

**Figure 1:**
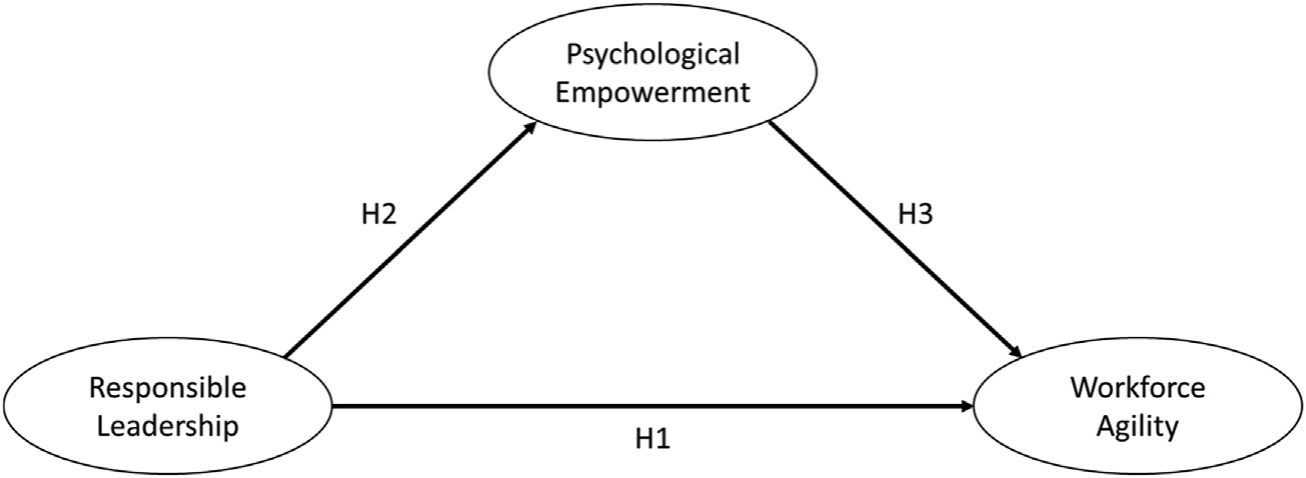
Conceptual framework of this study. Source: own study.

Figure 1 illustrates the research model used in the study. To test the hypotheses, we used scales verified in previous studies: to assess Responsible Leadership we used the Responsible Leadership Scale [38], to assess Workforce Agility we used the Workforce Agile Scale [33], and to assess Psychological Empowerment we used the Psychological Empowerment Scale [42].

## 3 Methods

### 3.1 Sampling

The study used a questionnaire-based survey as it is considered to be an efficient and effective way to collect data, especially when the research constructs have already been established and are well-known, as relevant data for these constructs can easily be obtained [56]. This study was approved by the Research Ethics Committee of Poznan University of Economics and Business. All participants were informed about the purpose of the study and voluntary consent was obtained. Participants’ anonymity was maintained and they had the freedom to discontinue their participation at any step of the survey. Participants were asked to complete a five-part questionnaire which included the following items: (1) Company information such as year established, size of workforce, energy market segment, area of activity and type of capital; (2) The Responsible Leadership Scale; (3) The Workforce Agile Scale; (4) The Psychological Empowerment Scale; and (5) Control Variables (position in the organisational hierarchy, managerial tenure, age, education and gender).

Through a professional research company (DRB Polonia), direct interviews (using CATI method) were conducted on a group of managers and experts working in 94 energy sector firms. Sample was limited to the employees of enterprises which hires more than 10 persons. The targeted sample size of this study was 187, as in similar studies [24]. The structure of the sample (taking into account the size of the workforce in the enterprises) is presented in Table 2.

**Table 2:** Characteristics of the surveyed.

Size of enterprise (number of employees)	% of respondents
10–49	53%
50–249	25%
250 and above	22%

The sample includes 15% high-level managers, 29% middle-level managers and 56% low-level managers or experts. Most of respondents (54%) had less than 10 years of managerial/expert experience, 29% had over 10 and less than 20 years of tenure, 17% had worked for over 20 years. Among the respondents, 35% had a secondary or vocational education, 26% had a bachelor's degree, 36% had a master's degree, and just 2% had a PhD. The majority of the sample were male (72%), and only 28% of the sample were female.

### 3.2 Measures

In the study we used a set of standardized questionnaires developed by Voegtlin [38], Breu et al. [33] and by Spreitzer [42], as previous research provided evidence of these scales criterion validity and the reliability [9, 10, 27, 35, 36, 58, 59, 60, 61]. All measurement scales were presented in Polish and English. The scales of this study were measured with a five-point Likert response format ranging from ‘1 = Not at all; Very strongly disagree’ to ‘5 = Frequently, if not always; Very strongly agree.’ Each part of the questionnaire is described below.

#### 3.2.1 Responsible leadership

Responsible leadership was assessed by participants using a scale developed by Voegtlin [38] (Table 3).

**Table 3:** Components of responsible leadership.

Symbol	Item name
RL1	Awareness of relevant stakeholder claims
RL2	Awareness of consequences of decisions for the affected stakeholders
RL3	Involving the affected stakeholders in the decision making process
RL4	Taking into account different stakeholder claims before making a decision
RL5	Achieving a consensus among the affected stakeholders

Source [38].

Sample items are “My direct supervisor weighs different stakeholder claims before making a decision”, “My direct supervisor tries to achieve a consensus among the affected stakeholders”. In order to enable the respondents to clearly understand the concept of stakeholders, we defined this construct in the questionnaire assuming, following Freeman, that a stakeholder may be any individual or group of individuals either affected by the company or who may be able to impact on the achievement of its objectives [62]. The scale ranges from 1 = Not at all to 5 = Frequently, if not always.

#### 3.2.2 Workforce agility

Workforce agility was assessed by participants using the 10-item modified scale developed by Breu et al. [33]. We modified the Breu et al. scale as we were referring not to information systems but to management systems in general. Therefore, we adopted the four dimensions of intelligence proposed by Breu et al. (sample statement is: “Our team is responsive to changing customer needs”), competencies (sample statement: “Our team can quickly develop new skills and competences”), collaboration (sample statement: “Our team's effectiveness of cooperating across functional boundaries is high”), culture (sample statement: “The employees on our team are empowered for independent decision making”), and instead of information systems, we adopted management systems (sample statement: “Our team is supported in introducing new management methods and techniques”). The scale ranges from 1 = Very Strongly Disagree to 5 = Very Strongly Agree. All the measurement items in the construct proposed by Breu et al. (both original and modified ones) are listed in Table 4.

**Table 4:** Components of workforce agility.

Symbol	Item name
WA1	Responsiveness to changing customer needs
WA2	Responsiveness to changing market conditions
WA3	Speed of developing new skills and competencies
WA4	Speed of acquiring the skills necessary for business process change
WA5	Speed of innovating management skills
WA6	Speed of acquiring new management skills
WA7	Effectiveness of cooperating across functional boundaries
WA8	Ease of moving between projects
WA9	Employee empowerment for independent decision making
WA10	Support for the introduction of new management systems and methods

Source [33].

#### 3.2.3 Psychological empowerment

Psychological empowerment was assessed by participants using a scale developed by Spreitzer [42], which comprises three items for each of the four subdimensions: meaning (a sample item is “My job activities are personally meaningful to me”), competence (a sample item is “I am self-assured about my capabilities to perform my work activities”), self-determination (a sample item is “I can decide on my own how to go about doing my work”), and impact (a sample item is “I have a great deal of control over what happens in my department”). The scale ranges from 1 = Very Strongly Disagree to 5 = Very Strongly Agree.

All the measurement items for the construct proposed by Spreitzer are listed in Table 5.

**Table 5:** Components of psychological empowerment.

Symbol	Item name	Dimension of psychological empowerment
PE1	Importance of work	Meaning
PE2	Meaningfulness of job activities	
PE3	Meaningfulness of work	
PE4	Confidence in ability to perform job	Competence
PE5	Self-assurance about capabilities to perform work activities	
PE6	Mastery of skills necessary for job	
PE7	Autonomy in determining job execution	Self-determination
PE8	Deciding on behaviour at work	
PE9	Independence and freedom at work	
PE10	Impact on the team	Impact
PE11	Control over the team	
PE12	Influence over the team	

Source [42].

#### 3.2.4 Control variables

The following control (demographic) variables were included in the study as additional variables: position in the organisational hierarchy, managerial tenure, age, education and gender [63]. Position in the organisational hierarchy was measured using three categories (1 = high level manager, 2 = middle level manager; 3 = low level manager or expert). Managerial tenure was measured using four categories (1 = under 10 years; 2 = 11–20 years; 3 = 21–30 years; 4 = 31 and more). Age was measured using three categories (1 = under 25; 2 = 26–49; 3–50 or older). Education was measured using four categories (1 = secondary or vocational education; 2 = bachelor; 3 = MBA; 4 = PhD). Gender was measured using two categories (1 = female, 2 = male). Due to the limited sample, the other category was removed. In a SEM analysis all variables were standardized.

Control variables were included in the model, except for gender, due to the weak scale in this case and the inability to assign ordering values. A conceptual diagram in which the influence of control variables on variables in the base model was included is presented in Figure 7.

### 3.3 Data analysis

*AMOS IBM SPSS Statistica* software was used to analyse the data. As the proposed model includes mediating variables and contains latent constructs that are being measured with multiple indicators, it was decided to use Structural equation modelling (SEM).

## 4 Results

In order to determine whether and how responsible leadership and psychological empowerment support workforce agility in the energy sector firms synthetic indicators were created based on the arithmetic mean of individual components.

Internal consistency of the responsible leadership scale was determined using Cronbach's alpha, the value of which in this case was 0.852.

**Figure 2:**
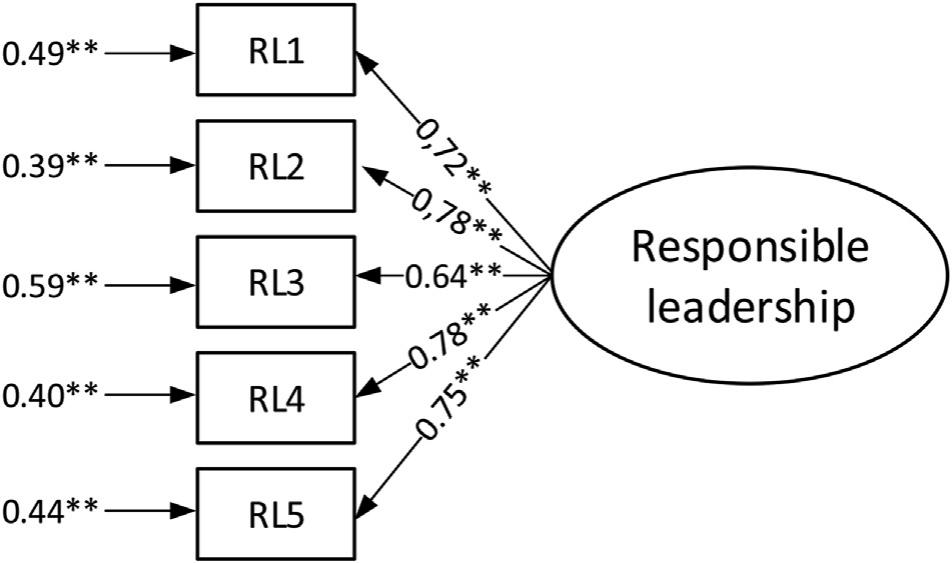
Confirmatory factor analysis for the responsible leadership measurement model (loadings and error variance). ∗∗p value < 0.01.

In order to confirm the reliability of the construct, confirmatory factor analysis was carried out (Figure 2).

For the responsible leadership measurement model, the results of the confirmatory analysis show high values of factor loading (all above 0.71) and composite reliability (0.853), while the SRMR, GFI, CFI, and IFI values were respectively: 0.034, 0.966, 0.972 and 0.972. This confirmed the convergent validity and the reliability of the construct and a good fit to the correlation matrix.

**Figure 3:**
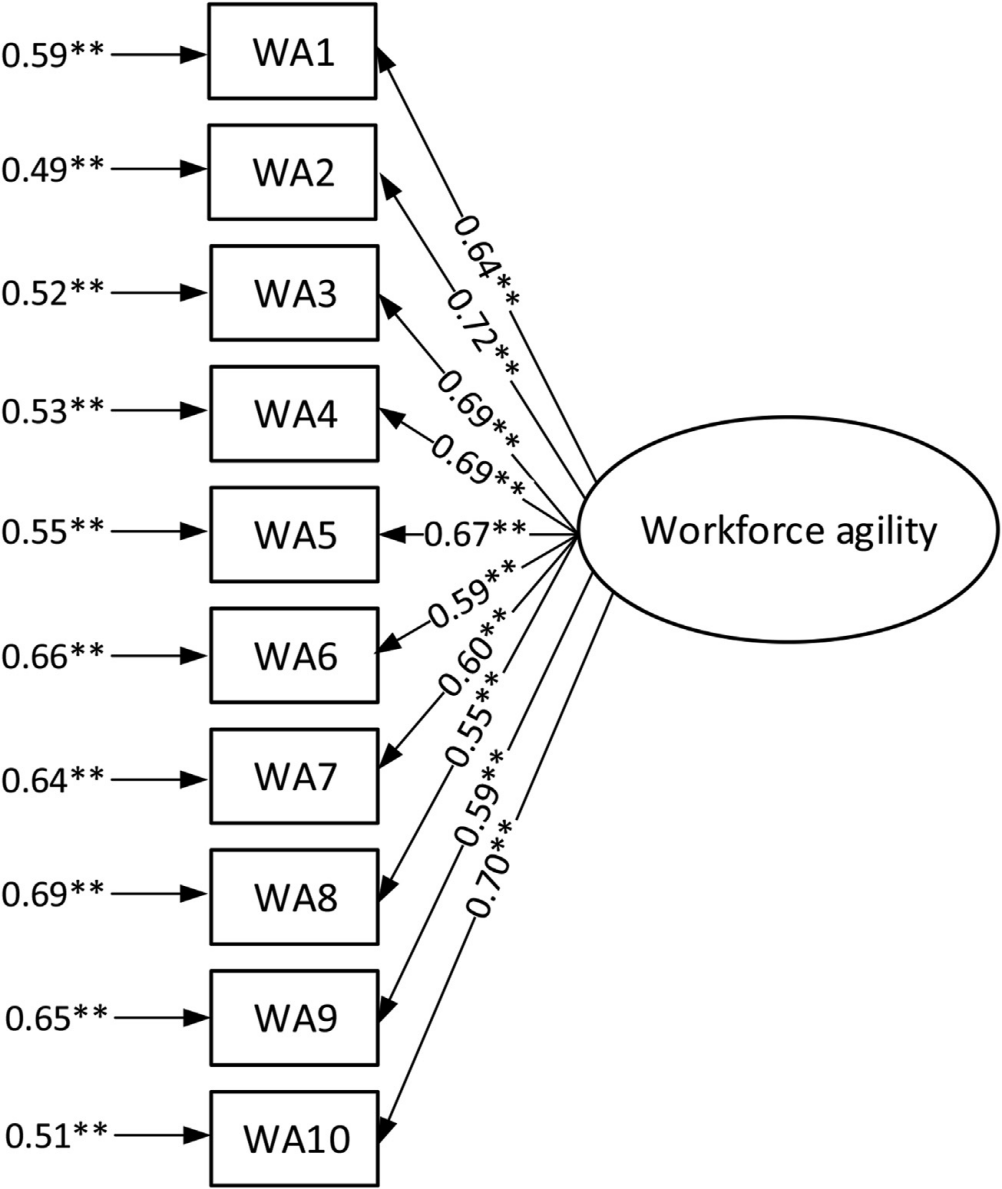
Confirmatory factor analysis for workforce agility index. ∗∗p value < 0.01.

Cronbach's alpha was calculated for the modified Breu et al. workforce agility scale, with the value achieved being 0.876. Also, confirmatory analysis was conducted, the results of which are shown in Figure 3.

Factor loading was above 0.55, which confirmed the convergent validity, and the composite reliability value (0.877) confirmed the reliability of the construct. For this model, the SRMR, GFI, CFI, and IFI values were respectively: 0.069, 0.868, 0.846, 0.848.

The values of the goodness of fit show that the fit of the model to the correlation matrix could be better (the values of GFI, CFI and IFI are below the preferred norms).

In order to obtain a better fit for the model, two paths with the lowest factor loading values and highest error value were removed. This meant removing the indicator components, which are: “speed of acquiring new management skills” (WA6) and “ease of moving between projects” (WA8).

**Figure 4:**
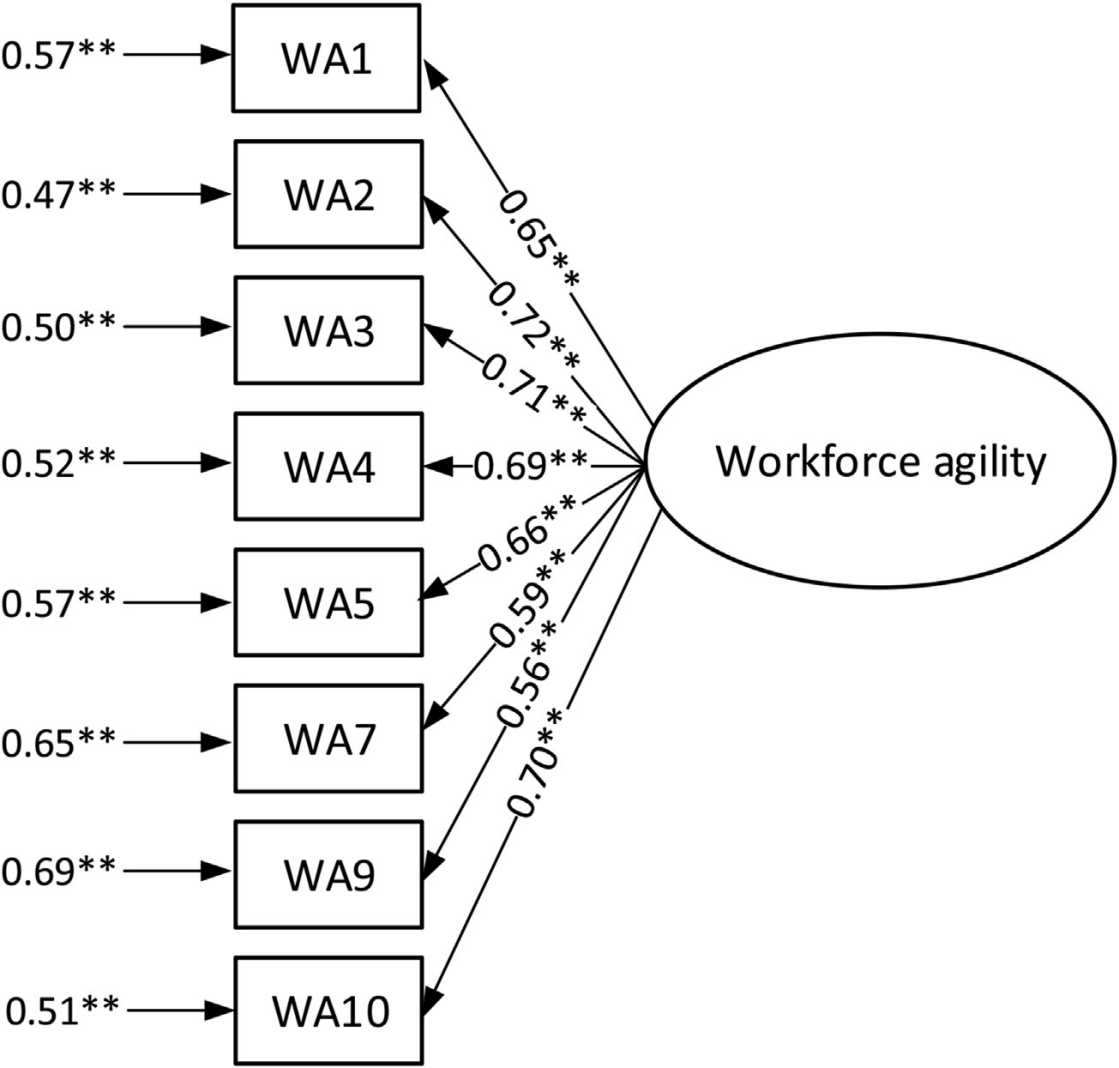
Confirmatory factor analysis for modified workforce agility index. ∗∗p value < 0.01.

Confirmatory factor analysis for the modified workforce agility index is shown in Figure 4.

For the modified workforce agility index, the composite reliability and Cronbach's alpha values were respectively 0.860 and 0.862. In this way, the values of the SRMR (0.059), GFI (0.910), CFI (0.906), and IFI (0.907) show that the model fits well with the data and empirical values.

For each of the dimensions proposed by Spreitzer, Cronbach's alpha was calculated (Table 6).

**Table 6:** Cronbach's alpha values for indicators of dimensions of psychological empowerment.

Indicator of dimension	Cronbach's alpha value
Meaning	0.848
Competence	0.851
Self-determination	0.818
Impact	0.844

**Figure 5:**
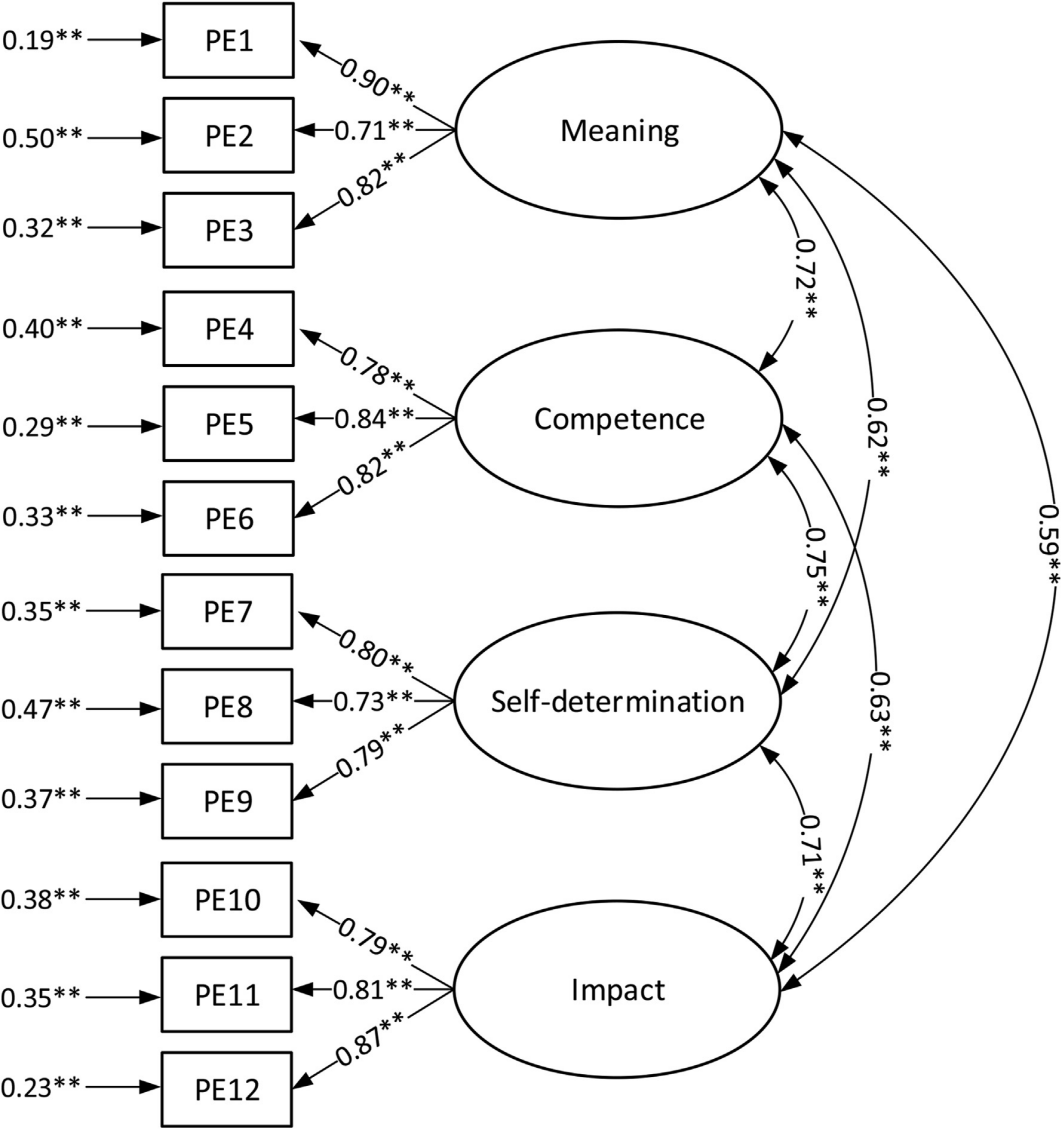
Confirmatory factor analysis for psychological empowerment index. ∗∗p value < 0.01.

The reliability of the construct was confirmed using confirmatory factor analysis (Figure 5).

A factor loading of above 0.71 and a composite reliability value of 0.957 confirmed the convergent validity and reliability of the construct. For this model, the SRMR, GFI, CFI and IFI values were respectively: 0.043, 0.918, 0.961 and 0.961.

**Figure 6:**
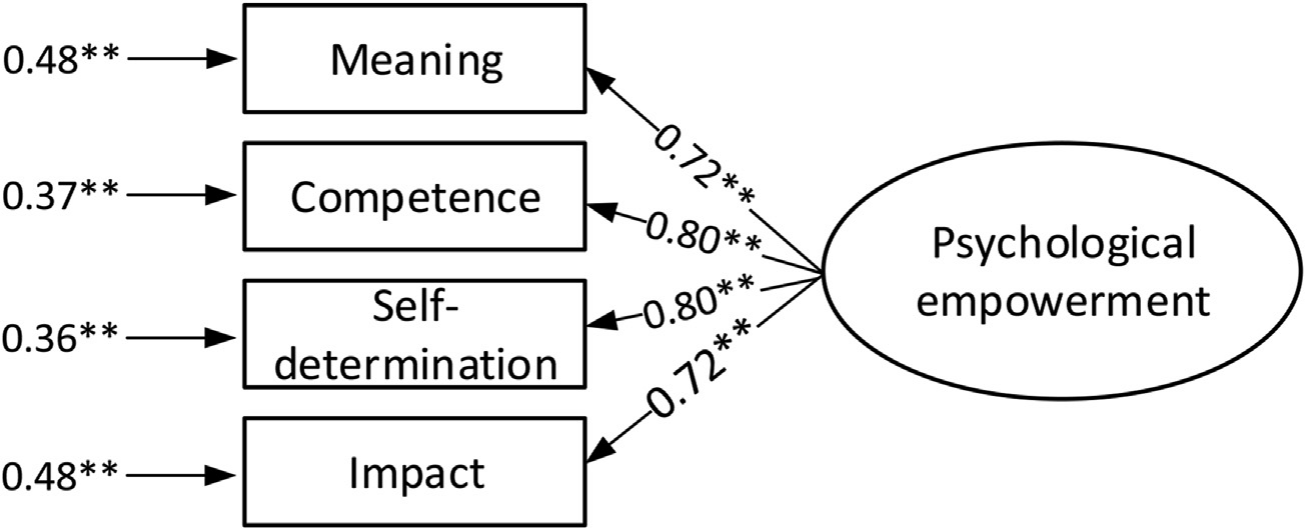
Confirmatory factor analysis for higher order psychological empowerment index. ∗∗p value < 0.01.

The values of the correlation coefficients between the indicators of psychological empowerment in the confirmatory analysis indicate that it is viable to describe this phenomena using one indicator. As a consequence, in the next step such an indicator was calculated (based on the average value of the dimension indicators). The results of confirmatory factor analysis of the measurement model for psychological empowerment is presented in Figure 6. For this analysis, the SRMR, GFI, CFI and IFI values were respectively: 0.020, 0.989, 0.993 and 0.993.

**Figure 7:**
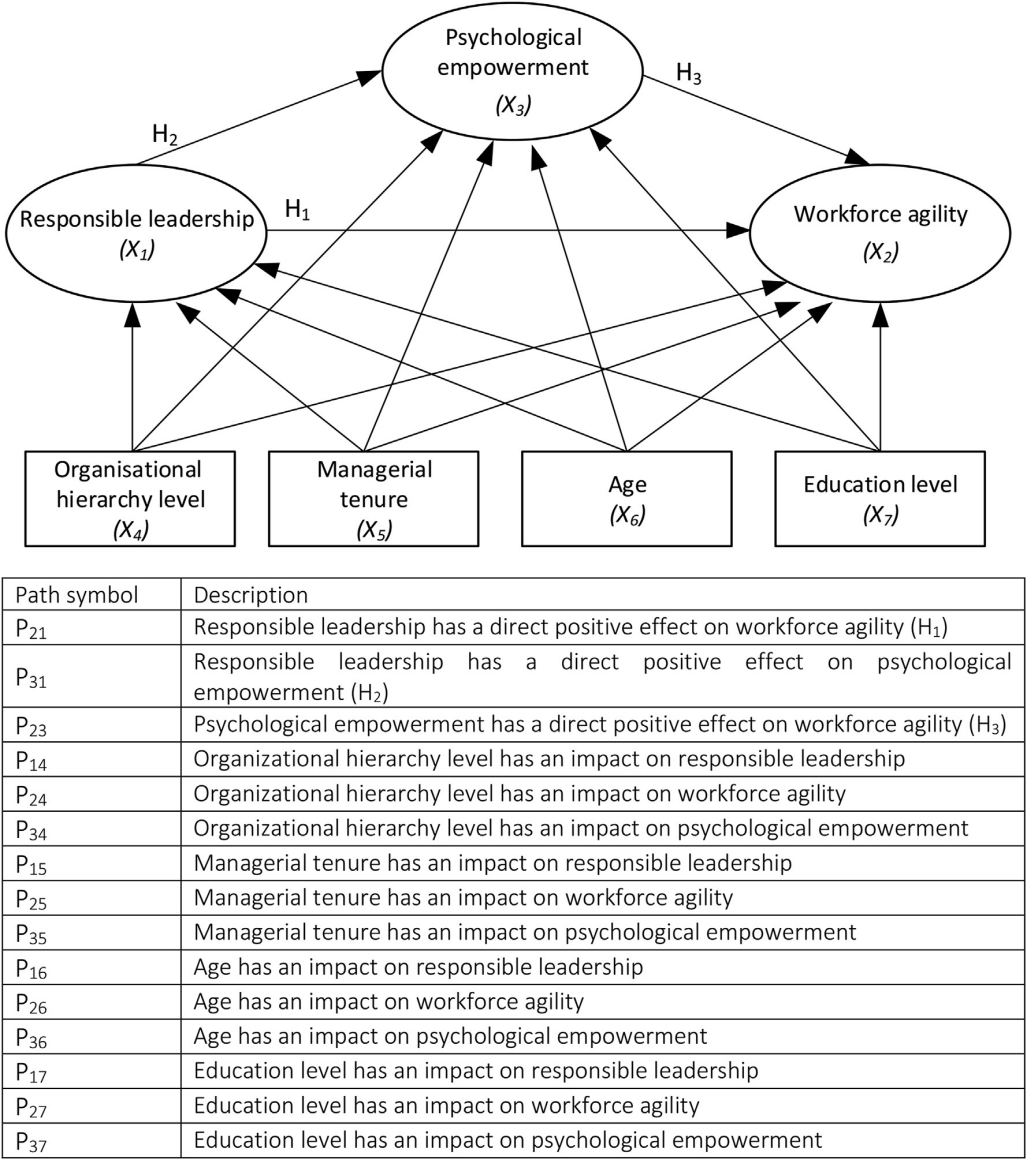
Conceptual diagram with control variables.

The results of the SEM analysis are shown in Table 7.

**Table 7:** Results of SEM analysis.

Path	Coefficient	p-value	t-value
P_21_	0.314	0.000	5.301
P_31_	0.489	0.000	8.871
P_23_	0.477	0.000	8.314
P_14_	0.073	0.338	0.958
P_24_	0.028	0.609	0.511
P_34_	0.017	0.792	0.264
P_15_	-0.012	0.887	-0.142
P_25_	-0.002	0.971	-0.036
P_35_	0.137	0.053	1.931
P_16_	-0.008	0.917	-0.105
P_26_	-0.058	0.319	-0.996
P_36_	-0.009	0.898	-0.128
P_17_	0.019	0.803	0.250
P_27_	-0.058	0.295	-1.047
P_37_	-0.174	0.006	-2.753
E_1_ ->X_1_	0.994	0.000	88.070
E_2_ ->X_2_	0.513	0.000	9.776
E_3_ ->X_3_	0.722	0.000	12.943

Due to the lack of degrees of freedom, it was impossible to calculate the fit indices for the given model. In the following steps, the paths whose parameters have the least statistically significant values have been, one by one, removed from the model (see Table 8). Removing first path already made it possible to calculate fit indexes.

**Table 8:** Steps of eliminating paths in SEM model.

Version	The difference to the initial/preceding model (paths that been rejected)	Fit index value				SRMR	p-value
		GFI	AGFI	NFI	CFI		
i+1	P_25_ = −0.002, (p-value = 0.971)	1.000	1.000	1.000	1.000	0.000	0.971
i+2	P_16_ = −0.008 (p-value = 0.917)	1.000	1.000	1.000	1.000	0.002	0.994
i+3	P_36_ = −0.009, (p-value = 0.898)	1.000	1.000	1.000	1.000	0.003	0.999
i+4	P_15_ = 0.016 (p-value = 0.837)	1.000	0.999	1.000	1.000	0.005	0.999
i+5	P_17_ = 0.017 (p-value = 0.819)	1.000	0.999	0.999	1.000	0.006	1.000
i+6	P_34_ = 0.018 (p-value = 0.786)	1.000	0.999	0.999	1.000	0.007	1.000
i+7	P_24_ = 0.028 (p-value = 0.606)	0.999	0.997	0.998	1.000	0.010	1.000
i+8	P_27_ = −0.052 (p-value = 0.333)	0.998	0.993	0.994	1.000	0.011	0.994
i+9	P_14_ = 0.074 (p-value = 0.312)	0.998	0.993	0.995	1.000	0.011	0.978
i+10	P_26_ = −0.063 (p-value = 0.234)	0.997	0.988	0.992	1.000	0.015	0.828

**Figure 8:**
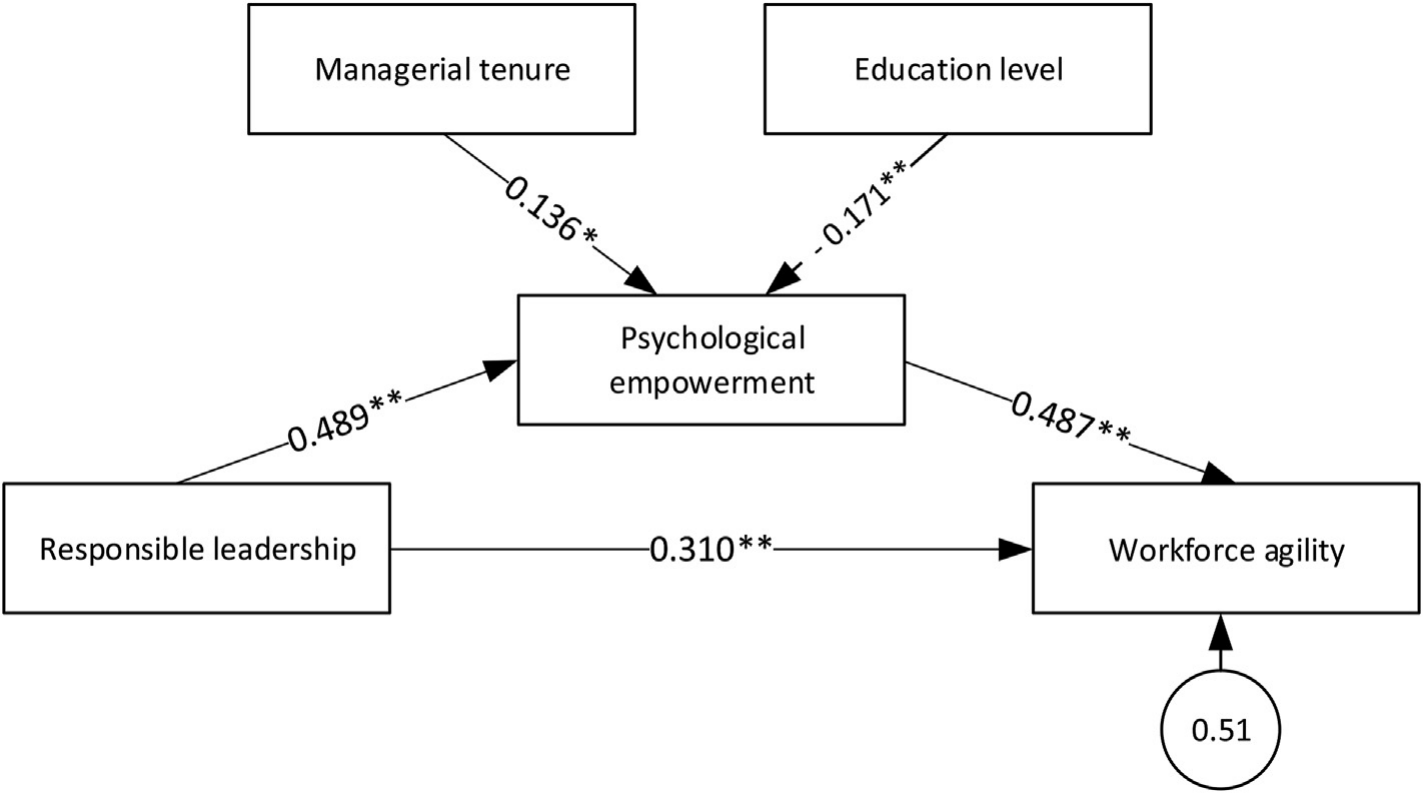
The final results of the SEM analysis. ∗p value < 0.05, ∗∗p value < 0.01.

The final results of the SEM analysis (shown in Figure 8) confirm all three hypothesis and also show that two control variables (managerial tenure and education level) has an impact of on psychological empowerment.

## 5 Discussion

This study focused on examining the relationship between responsible leadership, psychological empowerment, and workforce agility in the energy sector firms. The research results confirmed all the proposed hypotheses. First, confirmed that responsible leadership positively affects workforce agility (0.310, hypothesis H1). Dynamic changes in the energy sector prompted by environmental transformations and institutional pressure, make companies face a numerous challenges, such as complexity of technological innovation [1], fragmentation of markets [64] and increased customer expectations [4]. Assuming, as suggested by Abdola Bidhandi and Valmohammadi [7], that in unpredictable environments, agility becomes a basis of flexible organisational responses to environmental changes [7] our research goes beyond the assessment of technical factors and focuses on the workforce agility, recognising it, as Storme, as a foundation of organisational agility [32]. . In this aspect, we agree with the suggestions of Breu et al. [33] that in order to develop the overall company agility, organisations need to understand how to support the agility on the workforce level. Our findings suggesting that workforce agility is supported by responsible leadership are in line with the results of the Joiner and Petermann and Zacher studies Zacher [11, 15] that developing the workforce agility requires responsible leadership, which plays a significant role in empowering employees. Our results suggest that providing workforce agility in energy sector firms requires leadership that is involved in creating social capital and oriented towards developing sustainable business. We argue that responsible leadership can facilitate workshop agility through emphasis on relations with stakeholders. These relations, as showed by Sarkar [28], need flexibility as stakeholders’ expectations change. In this area, the results of our research confirm findings recommending CEOs to develop a proactive practice in leading organisational change, managing teams and conducting key interviews [11], as well as conclusions showing that leadership plays a significant role in initiating cooperation between teams and departments, as well as in empowering employees to create an agile culture [15]. Our results support the recommendations relating to the advisability to develop proactive practices in leading organisational change, managing teams and conducting key interviews [11] and suggest that leadership should play a significant role in initiating cooperation between teams and departments and empowering employees to create an agile culture [15].

Our results, confirming the positive relation between responsible leadership and psychological empowerment (0.489, hypothesis H2), are consistent with Haque et al. [3] observations suggesting a positive influence of responsible leadership on the organisational commitment of employees, as well as Maak and Pless findings [2] showing, that responsible leaders should train and empower employees to achieve goals in an ethical, respectful, and ‘relatively intelligent’ manner by creating a system of incentives for respectful collaboration in order to support stakeholder responses. The results of our research allow us to assume that the energy sector firms managers, by strengthening responsible leadership, could affect the sense of internal motivation related to the work environment, which could ensure the possibility of effective achievement of goals.

Our study, suggesting that the energy sector companies ensure an appropriate organisational agility level through workforce agility (0.487, hypothesis H3), is in line with the Sherehiy and Karwowski [1] and Almahamid findings [2] and is in opposition to the Malik et al. results (2021) pointing, that agile practices related to team diversity and incremental and iterative development do not have a valid relationship with psychological empowerment. This observation can be considered as important from the point of view of breaking resistance to change, in line with the Storme et al. suggestion [32] that one way to overcoming the resistance to changes is to understand the psychological characteristics of agile workers.

Finally, our study shows that the value of indirect influence of responsible leadership on the agility of the workforce, where the mediating variable is psychological empowerment, was 0.24. This coexists with a direct impact of responsible leadership on the agility of the workforce, which value is not so much higher (0.31).

## 6 Conclusion

In this paper, we have analysed whether and how responsible leadership and psychological empowerment drive workforce agility in the energy sector firms. Drawing from a discussion in the leadership theory, as well as agility and empowerment concepts, we developed a research model and formulated three hypotheses demonstrating the relationships between these constructs. Using Structural Equation Modelling, we identified the relationships between responsible leadership, psychological empowerment and workforce agility in energy sector firms. The results support the argument for the relationship between leadership focused on responsible management, psychological empowerment, and workforce agility. We found that a combination of responsible leadership and psychological empowerment influences the workforce agility.

The results of our research have direct theoretical implications. First, this study enriches the literature on responsible leadership theory by demonstrating that responsible leadership in the energy sector firms is an important variable influencing the workforce agility. Since some studies discuss a general relationship between leadership and workforce agility [65, 66], it is important to note that responsible leadership affects workforce agility by ensuring learning agility, flexibility and a higher level of stakeholder orientation. Second, we have discussed the impact of responsible leadership in the energy sector firms in combination with psychological empowerment on workforce agility. Scholarly studies have analysed the relationship between leadership and psychological empowerment [18, 45, 46, 47], with no direct linkages being found between responsible leadership and psychological empowerment. Thus, our paper fills a gap by pointing to the important link between responsible leadership and psychological empowerment. Third, our findings suggest the relationships between responsible leadership and psychological empowerment in the energy sector firms. Many studies have demonstrated the relationship between leadership and empowerment, but they mainly describe different types of leadership. There is no direct relationship found between variables describing responsible leadership and psychological empowerment. In this sense, our paper fills a gap by pointing to the important link between responsible leadership and psychological empowerment. Fourth, we have argued that psychological empowerment is related to workforce agility, and our study has showed that psychological empowerment plays an important role in stimulating workforce agility. This observation is consistent with the findings Muduli and Pandya show that psychological empowerment, namely meaningfulness, competence, self-determination and impact, influence workforce agility [10].

The research results have some practical implications, which are significant with regard to actions taken by companies in order to adapt to the changing environment in the energy sector. The first category is to develop responsible leadership competences that allow management in the energy sector firms to respond to unexpected environmental changes, identify and exploit opportunities, and to manage crisis situations. This category (developing responsible leadership) is important in ensuring the development of energy sector companies. The second category is empowering employees. The third category is stimulating workforce agility to make sure that the whole organisation will be able to respond efficiently to changes in an unpredictable, dynamic and hostile environment.

This study has limitations which may imply some possible future research. First, the study was conducted on a limited sample of respondents from energy sector companies, varied in terms of age and size, which made it difficult to draw universal conclusions. In the future research, it would be worth increasing the research sample and limiting the respondents to those from medium-sized enterprises, to provide valuable insights into their potential for harnessing workforce agility. Second, when planning the survey, we used existing, validated measurement scales. We are aware that the list of measurements developed for responsible leadership, empowerment and workforce agility may not be exhaustive or complete. Therefore, in the future research, it would be worth expanding the studied variables, in particular regarding the relationship between psychological empowerment and workforce agility.

## Declarations

### Author contribution statement

All authors listed have significantly contributed to the development and the writing of this article.

### Funding statement

This research did not receive any specific grant from funding agencies in the public, commercial, or not-for-profit sectors.

### Data availability statement

Data will be made available on request.

### Declaration of interest’s statement

The authors declare no conflict of interest.

### Additional information

No additional information is available for this paper.

## References

[1] Baloch M.A., Ozturk I., Bekun F.V., Khan D. (2021). Modeling the dynamic linkage between financial development, energy innovation, and environmental quality: does globalization matter?. Bus. Strat. Environ..

[2] D’Haeseleer W., De Vries L., Kang C., Delarue E. (2017). Flexibility challenges for energy markets: fragmented policies and regulations lead to significant concerns. IEEE Power Energy Mag..

[3] Latapí Agudelo M.A., Johannsdottir L., Davidsdottir B. (2020). Drivers that motivate energy companies to be responsible. A systematic literature review of Corporate Social Responsibility in the energy sector. J. Clean. Prod..

[4] Heinen S., Richards P. (2020). Towards customer-centric energy utilities - a granular data-driven bottom-up approach to understanding energy customer trends. Electr. J..

[5] Soutar I. (2021). Dancing with complexity: making sense of decarbonisation, decentralisation, digitalisation and democratisation. Energy Res. Social Sci..

[6] Schaeffer G.J. (2015). Energy sector in transformation, trends and prospects. Procedia Comput. Sci..

[7] Abdoli Bidhandi R., Valmohammadi C. (2017). Effects of supply chain agility on profitability. Bus. Process Manag. J..

[8] Al-Faouri A.H., Al-Nsour M.M., Al-Kasasbeh M.M. (2014). The impact of workforce agility on organizational memory. Knowl. Manag. Res. Pract..

[9] Muduli A. (2017). Workforce agility: examining the role of organizational practices and psychological empowerment. Glob. Bus. Organ. Excell..

[10] Muduli A., Pandya G. (2018). Psychological empowerment and workforce agility. Psychol. Stud..

[11] Joiner B. (2019). Leadership agility for organizational agility. J. Creat. Value.

[12] Liu H.M. (2020). Effect of partnership quality on SMEs success: mediating role of coordination capability and organisational agility. Total Qual. Manag. Bus. Excell..

[13] Sherehiy B., Karwowski W. (2014). The relationship between work organization and workforce agility in small manufacturing enterprises. Int. J. Ind. Ergon..

[14] Paul M., Jena L.K., Sahoo K. (2020). Workplace spirituality and workforce agility: a psychological exploration among teaching professionals. J. Relig. Health.

[15] Petermann M.K.H., Zacher H. (2021). Development of a behavioral taxonomy of agility in the workplace. Int. J. Manag. Proj. Bus..

[16] Varshney D., Varshney N.K. (2020). Workforce agility and its links to emotional intelligence and workforce performance: a study of small entrepreneurial firms in India. Glob. Bus. Organ. Excell..

[17] Godkin L. (2015). Mid-management, employee engagement, and the generation of reliable sustainable corporate social responsibility. J. Bus. Ethics.

[18] Kundu S.C., Kumar S., Gahlawat N. (2019). Empowering leadership and job performance: mediating role of psychological empowerment. Manag. Res. Rev..

[19] Li H., Shi Y., Li Y., Xing Z., Wang S., Ying J., Zhang M., Sun J. (2018). Relationship between nurse psychological empowerment and job satisfaction: a systematic review and meta-analysis. J. Adv. Nurs..

[20] Laschinger H.K.S., Finegan J.E., Shamian J., Wilk P. (2004). A longitudinal analysis of the impact of workplace empowerment on work satisfaction. J. Organ. Behav..

[21] Speer P.W., Peterson N.A., Armstead T.L., Allen C.T. (2013). The influence of participation, gender and organizational sense of community on psychological empowerment: the moderating effects of income. Am. J. Community Psychol..

[22] Ayala Calvo J.C., García G.M. (2018). Hardiness as moderator of the relationship between structural and psychological empowerment on burnout in middle managers. J. Occup. Organ. Psychol..

[23] Pradhan R.K., Panda M., Jena L.K. (2017). Transformational leadership and psychological empowerment: the mediating effect of organizational culture in Indian retail industry. J. Enterprise Inf. Manag..

[24] Haque A., Fernando M., Caputi P. (2019). The relationship between responsible leadership and organisational commitment and the mediating effect of employee turnover intentions: an empirical study with Australian employees. J. Bus. Ethics.

[25] Maak T., Pless N.M., Ethics J. Bus. (2006). Responsible Leadership in a Stakeholder Society – A Relational Perspective.

[26] Maak T., Pless N.M., Voegtlin C. (2016). Business statesman or shareholder advocate? CEO responsible leadership styles and the micro-foundations of political CSR. J. Manag. Stud..

[27] Javed M., Ali H.Y., Asrar-ul-Haq M., Ali M., Kirmani S.A.A. (2020). Responsible leadership and triple-bottom-line performance—do corporate reputation and innovation mediate this relationship?. Leader. Organ. Dev. J..

[28] Sarkar A. (2016). We live in a VUCA World: the importance of responsible leadership. Dev. Learn. Organ..

[29] Maak T. (2007). Responsible leadership, stakeholder engagement, and the emergence of social capital. J. Bus. Ethics.

[30] Miska C., Hilbe C., Mayer S. (2014). Reconciling different views on responsible leadership: a rationality-based approach. J. Bus. Ethics.

[31] Voegtlin C., Patzer M., Scherer A.G. (2012). Responsible leadership in global business: a new approach to leadership and its multi-level outcomes. J. Bus. Ethics.

[32] Storme M., Suleyman O., Gotlib M., Lubart T. (2020). Who is agile? An investigation of the psychological antecedents of workforce agility. Glob. Bus. Organ. Excell..

[33] Breu K., Hemingway C.J., Strathern M., Bridger D. (2002). Workforce agility: the new employee strategy for the knowledge economy. J. Inf. Technol..

[34] Qin R., Nembhard D.A. (2015). Workforce agility in operations management. Surv. Oper. Res. Manag. Sci..

[35] Uner S., Turan S. (2010). The construct validity and reliability of the Turkish version of Spreitzer’s psychological empowerment scale. BMC Publ. Health.

[36] Siegall M., Gardner S. (2000). Contextual factors of psychological empowerment. Person. Rev..

[37] Sun N., Li Q.J., Lv D.M., Lin P., Lu G.Z., An X.M. (2011). The psychometric properties of the Chinese version of the problem areas in psychological empowerment scale (PES): scale development. J. Clin. Nurs..

[38] Voegtlin C. (2011). Responsible Leadersh..

[39] Doh J.P., Stumpf S.A., Tymon W.G. (2012). Responsible leadership helps retain talent in India. Responsible Leadersh.

[40] Stewart J.G., McNulty R., Griffin M.T.Q., Fitzpatrick J.J. (2010). Psychological empowerment and structural empowerment among nurse practitioners. J. Am. Acad. Nurse Pract..

[41] Thomas K.W., Velthouse B.A. (1990). Cognitive elements of empowerment: an “interpretive” model of intrinsic task motivation. Acad. Manag. Rev..

[42] Spreitzer G.M. (1995). Psychological empowerment in the workplace: dimensions, measurement, and validation. Acad. Manag. J..

[43] Doh J.P., Quigley N.R. (2014). Acad. Manag. Perspect..

[44] Alavi S., Wahab D. Abd., Muhamad N., Arbab Shirani B. (2014). Organic structure and organisational learning as the main antecedents of workforce agility. Int. J. Prod. Res..

[45] Javed B., Khan A.A., Bashir S., Arjoon S. (2017). Impact of ethical leadership on creativity: the role of psychological empowerment. Curr. Issues Tourism.

[46] Suifan T.S., Diab H., Alhyari S., Sweis R.J. (2020). Does ethical leadership reduce turnover intention? The mediating effects of psychological empowerment and organizational identification. J. Hum. Behav. Soc. Environ..

[47] Grošelj M., Černe M., Penger S., Grah B. (2020). Authentic and transformational leadership and innovative work behaviour: the moderating role of psychological empowerment. Eur. J. Innovat. Manag..

[48] Houghton J.D., Yoho S.K. (2005). Toward a contingency model of leadership and psychological empowerment: when should self-leadership Be encouraged?. J. Leader. Organ Stud..

[49] Van Der Wagen M. (2020). Leadership and motivation. Cust. Serv. Intell..

[50] Zhou J., George J.M. (2003). Awakening employee creativity: the role of leader emotional intelligence. Leader. Q..

[51] Kim M., Beehr T.A., Prewett M.S. (2018). Employee responses to empowering leadership: a meta-analysis. J. Leader. Organ Stud..

[52] Antunes A., Franco M. (2016). How people in organizations make sense of responsible leadership practices: multiple case studies. Leader. Organ. Dev. J..

[53] Waldman D.A., Galvin B. (2008). Alternative perspectives of responsible leadership. Organ. Dynam..

[54] Stone-Johnson C. (2014). Responsible leadership. Educ. Adm. Q..

[55] Muduli A. (2016). Exploring the facilitators and mediators of workforce agility: an empirical study. Manag. Res. Rev..

[56] Almahamid S. (2019). The influence of ERP system usage on agile capabilities: examining the mediating role of users’ psychological empowerment in Jordanian commercial banks. Inf. Technol. People.

[57] Malik M., Sarwar S., Orr S. (2021). Agile practices and performance: examining the role of psychological empowerment. Int. J. Proj. Manag..

[58] Javed M., Rashid M.A., Hussain G., Ali H.Y. (2020). The effects of corporate social responsibility on corporate reputation and firm financial performance: moderating role of responsible leadership. Corp. Soc. Responsib. Environ. Manag..

[59] Han Z., Wang Q., Yan X. (2019). How responsible leadership predicts organizational citizenship behavior for the environment in China. Leader. Organ. Dev. J..

[60] Raut P.K., Das J.R., Gochhayat J., Das K.P. (2022). Influence of workforce agility on crisis management: role of job characteristics and higher administrative support in public administration. Mater. Today Proc..

[61] Stewart J.G., McNulty R., Griffin M.T.Q., Fitzpatrick J.J. (2010). Psychological empowerment and structural empowerment among nurse practitioners. J. Am. Acad. Nurse Pract..

[62] Freeman R.E. (1984).

[63] Bernerth J.B., Aguinis H. (2016). A critical review and best-practice recommendations for control variable usage. Person. Psychol..

[64] Andoni M., Robu V., Flynn D., Abram S., Geach D., Jenkins D., McCallum P., Peacock A. (2019). Blockchain technology in the energy sector: a systematic review of challenges and opportunities. Renew. Sustain. Energy Rev..

[65] Ajgaonkar S., Neelam N.G., Wiemann J. (2021). Drivers of workforce agility: a dynamic capability perspective. Int. J. Organ. Anal..

[66] Parker D.W., Holesgrove M., Pathak R. (2015). Improving productivity with self-organised teams and agile leadership. Int. J. Prod. Perform. Manag..

